# Malignant Lymphoepithelioma of the Submandibular Gland: a rare entity

**DOI:** 10.1590/S1808-86942010000600023

**Published:** 2015-10-19

**Authors:** Francisco Júlio Muniz Neto, José Alberto Alves Oliveira, Conceição Aparecida Machado de Souza Campos, Luis Alberto Albano Ferreira

**Affiliations:** 1 Higher education, fifth-year medical student, Medical School of the Ceará State University; 2 Higher education, fifth-year medical student, Medical School of the Ceará State University. Science initiation bursary (FUNCAP); 3 Graduation course (master's degree), staff physician of the Radiotherapy Unit of the Ceará Cancer Institute / Ceará Cancer Hospital. Master's student in the Dinter-Minter program; 4 Graduation course, medical surgeon of the Walter Cantídio Hospital / Ceará Federal University, and the Albert Sabin Child Hospital / Health Secretariat, Ceará State. This study was carried out at the Head & Neck Surgery Unit and the Radiotherapy Unit of the Ceará Cancer Institute, Fortaleza, CE, Brazil

**Keywords:** carcinoma, submandibular gland, radiotherapy

## INTRODUCTION

Salivary gland tumors are relatively rare, comprising about 3 to 4% of all neck and face tumors. The malignant lymphoepithelial lesion (MLEL) is a variety of the planocellular type, in which intense lymphoid infiltration is found in its fibrous stroma. There is a 3:2 female to male incidence of MLEL, except among the Chinese, in which the tumor is more prevalent in males.^1^

The MLEL involves only salivary glands, generally the parotid gland; about 15% of cases occur in the submandibular gland.^2^ The pathogenesis has been related to the Epstein-Barr virus (EBV), because of the detection of viral oncoproteins in tumor cell cultures. After an infection, the virus may remain at a low level of activity for a prolonged period in the epithelial salivary ducts, and in a latent state in B2 lymphocytes.

The EBV has been correlated with several epithelial malignancies, especially undifferentiated nasopharyngeal carcinomas (NPC). Recent studies have also suggested a strong association between EBV expression and the MLEL in salivary glands among the Taiwanese, South China Chinese, and Eskimos.^3^

Patients with the MLEL are apparently more prone to regional and distance metastases. The incidence of regional metastases among Eskimos is 30% to 50%; among Asians, the incidence is 10%.^1^

## CASE REPORT

A female white patient aged 75 years presented with a right submandibular node on October 2006. Ultrasound and fine-needle aspiration puncture were done; the latter was negative for neoplastic cells. The right salivary gland was partially removed on December 2006.

The patient was referred to an oncology unit on January 2007, since histology of the surgical specimen diagnosed an undifferentiated submandibular gland lymphoepithelioma-like carcinoma. The local and regional examination revealed a surgical scar on the right submandibular region; there were no palpable neck nodes, and examination of the mouth and nasofibroscopy were normal. A review of the slides confirmed the diagnosis. Immunohistochemistry also suggested a lymphoepithelial carcinoma (WHO) with positive cytokeratins 40, 48, 50 and 50.6 kDa. Face and neck computed tomography did not reveal enlarged lymph nodes or lesions in the rhinopharynx.

The initial treatment consisted of alloy collimated electron radiotherapy on the right field over the entire operated area and the first drainage level. The initial dose was 5,000 cGy, 12 MeV, 25 fractions (5 weekly fractions) followed by 1,000 cGy reinforcement in 5 fractions to reach 6,000 cGy; the radiotherapy period was 23 February 2007 to 13 April 2007.

**Figure 1:**
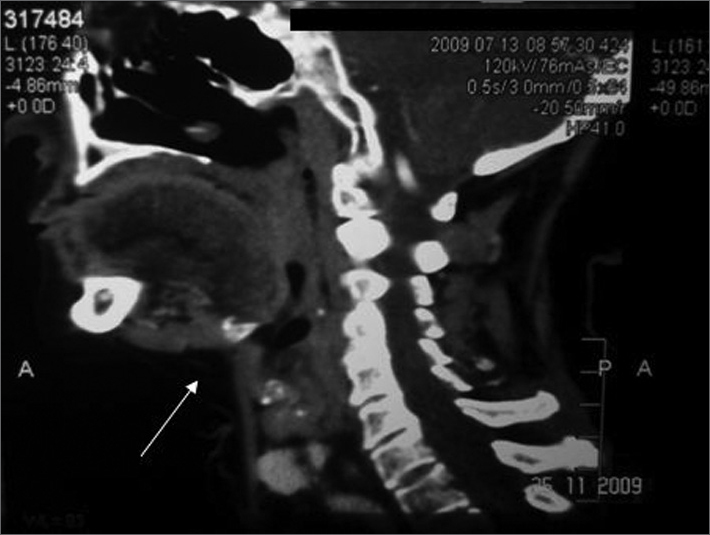
Operated site with no local or regional recurrences.

At present, the patient has no evidence of local or regional recurrences; the irradiated area is not fibrotic. Imaging demonstrates absence of the right submandibular gland (Fig. 1).

## DISCUSSION

The nasopharynx, the salivary glands and the thymus derive from the primitive pharynx. Undifferentiated carcinomas in these sites are histologically identical.^1^ Thus, NPC are indiscernible.

It is important to differentiate the MLEL from NPC metastases to salivary glands. If the diagnosis is incorrect, the NPC will not receive the appropriate radiotherapy dose even if adjuvant therapy is done in salivary glands. Thus, to establish the diagnosis of the MLEL, careful nasofibroscopy of the upper respiratory tract and random biopsies of the nasopharynx should be carried out to discard metastatic disease.^1^

The MLEL tumors may be infiltrative with indistinct limits, partially circumscribed, or multinodular. Regional metastases first occur around the parotid gland, the superior region of the neck, the retroauricular lymph nodes; later, they involve supraclavicular and paratracheal lymph nodes.^1^ Computed tomography of the cranial base and nasopharynx should be carried out for the diagnosis, staging, and follow-up. Magnetic resonance imaging is better for evaluating recurrences and soft tissue involvement.^4^

Because of the malignant behavior of the lymphoepithelioma and the high metastatic rate to neck lymph nodes during the initial stages (stages I and II), neck dissection is recommended.^5^ Some studies have showed that complete surgical excision with free margins is mandatory.

Tumor removal with wide margins, neck dissection, and postoperative combined radiotherapy and chemotherapy comprise the treatment of the MLEL in patients at advanced stages of the disease (stages III and IV). Removal of the tumor and neck dissection, followed by postoperative radiotherapy, comprise the treatment for patients at initial stages of the disease (stages I and II). Salvage surgery and chemotherapy are recommended for patients with local or regional recurrences. Thus, surgeons should assess the tumor stage and to seek evidence of neck disease when defining the treatment.^5^

## COMMENTS

Because of the high recurrence rate and metastases of the MLEL, the ideal treatment should be aggressive; this includes extensive resection with tumor-free margins, dissection of neck lymph node metastases, and electron radiotherapy or radiotherapy and adjuvant chemotherapy when distance metastases are present.
